# Perinatal derivatives application: Identifying possibilities for clinical use

**DOI:** 10.3389/fbioe.2022.977590

**Published:** 2022-10-11

**Authors:** Florelle Gindraux, Nicola Hofmann, Marta Agudo-Barriuso, Mariastefania Antica, Pedro Silva Couto, Marie Dubus, Serhiy Forostyak, Lenart Girandon, Roberto Gramignoli, Marcin Jurga, Sergio Liarte, Ruta Navakauskiene, Volodymyr Shablii, Xavier Lafarge, Francisco J. Nicolás

**Affiliations:** ^1^ Service de Chirurgie Orthopédique, Traumatologique et Plastique, CHU- Besançon, France; ^2^ Laboratoire de Nanomédecine, Imagerie, Thérapeutique, Université Bourgogne Franche-Comté, Besançon, France; ^3^ German Society for Tissue Transplantation (DGFG) gGmbH, Hannover, Germany; ^4^ Experimental Ophthalmology Group, University of Murcia and Instituto Murciano de Investigación Biosanitaria (IMIB), Campus Ciencias de la Salud, Murcia, Spain; ^5^ Division of Molecular Biology, Rudjer Boskovic Institute, Zagreb, Croatia; ^6^ Department of Biochemical Engineering, Advanced Centre for Biochemical Engineering, University College London, London, United Kingdom; ^7^ Biomatériaux et Inflammation en Site Osseux (BIOS) EA 4691, Université de Reims Champagne Ardenne, Reims, France; ^8^ PrimeCell Bioscience, Ostrava, Czech Republic; ^9^ Department of Burns and Plastic Surgery, Faculty of Medicine, Institution Shared With University Hospital Brno, Masaryk University, Brno, Czechia; ^10^ Educell ltd, Trzin, Slovenia; ^11^ Department of Laboratory Medicine, Division of Pathology, Karolinska Institutet, Stockholm, Sweden; ^12^ EXO Biologics (NV), Liege, Belgium; ^13^ Regeneration, Molecular Oncology and TGFβ, IMIB-Arrixaca, Murcia, Spain; ^14^ Department of Molecular Cell Biology, Institute of Biochemistry, Life Sciences Center, Vilnius University, Vilnius, Lithuania; ^15^ Laboratory of Biosynthesis of Nucleic Acids, Institute of Molecular Biology and Genetics, Department of Functional Genomics, National Academy of Science, Kyiv, Ukraine; ^16^ Placenta Stem Cell Laboratory, Cryobank, Institute of Cell Therapy, Kyiv, Ukraine; ^17^ Établissement Français du Sang Nouvelle-Aquitaine, France/INSERM U1035, Université de Bordeaux, Biothérapie des Maladies Génétiques Inflammatoires et Cancers (BMGIC), Bordeaux, France

**Keywords:** clinical trials, perinatal derivatives, ICD-10 = international classification of diseases, questionnaire for PnD use in human conditions, amniotic membrane

## Abstract

Perinatal derivatives are drawing growing interest among the scientific community as an unrestricted source of multipotent stromal cells, stem cells, cellular soluble mediators, and biological matrices. They are useful for the treatment of diseases that currently have limited or no effective therapeutic options by means of developing regenerative approaches. In this paper, to generate a complete view of the state of the art, a comprehensive 10-years compilation of clinical-trial data with the common denominator of PnD usage has been discussed, including commercialized products. A set of criteria was delineated to challenge the 10-years compilation of clinical trials data. We focused our attention on several aspects including, but not limited to, treated disorders, minimal or substantial manipulation, route of administration, dosage, and frequency of application. Interestingly, a clear correlation of PnD products was observed within conditions, way of administration or dosage, suggesting there is a consolidated clinical practice approach for the use of PnD in medicine. No regulatory aspects could be read from the database since this information is not mandatory for registration. The database will be publicly available for consultation. In summary, the main aims of this position paper are to show possibilities for clinical application of PnD and propose an approach for clinical trial preparation and registration in a uniform and standardized way. For this purpose, a questionnaire was created compiling different sections that are relevant when starting a new clinical trial using PnD. More importantly, we want to bring the attention of the medical community to the perinatal products as a consolidated and efficient alternative for their use as a new standard of care in the clinical practice.

## 1 Introduction

**FIGURE 1:**
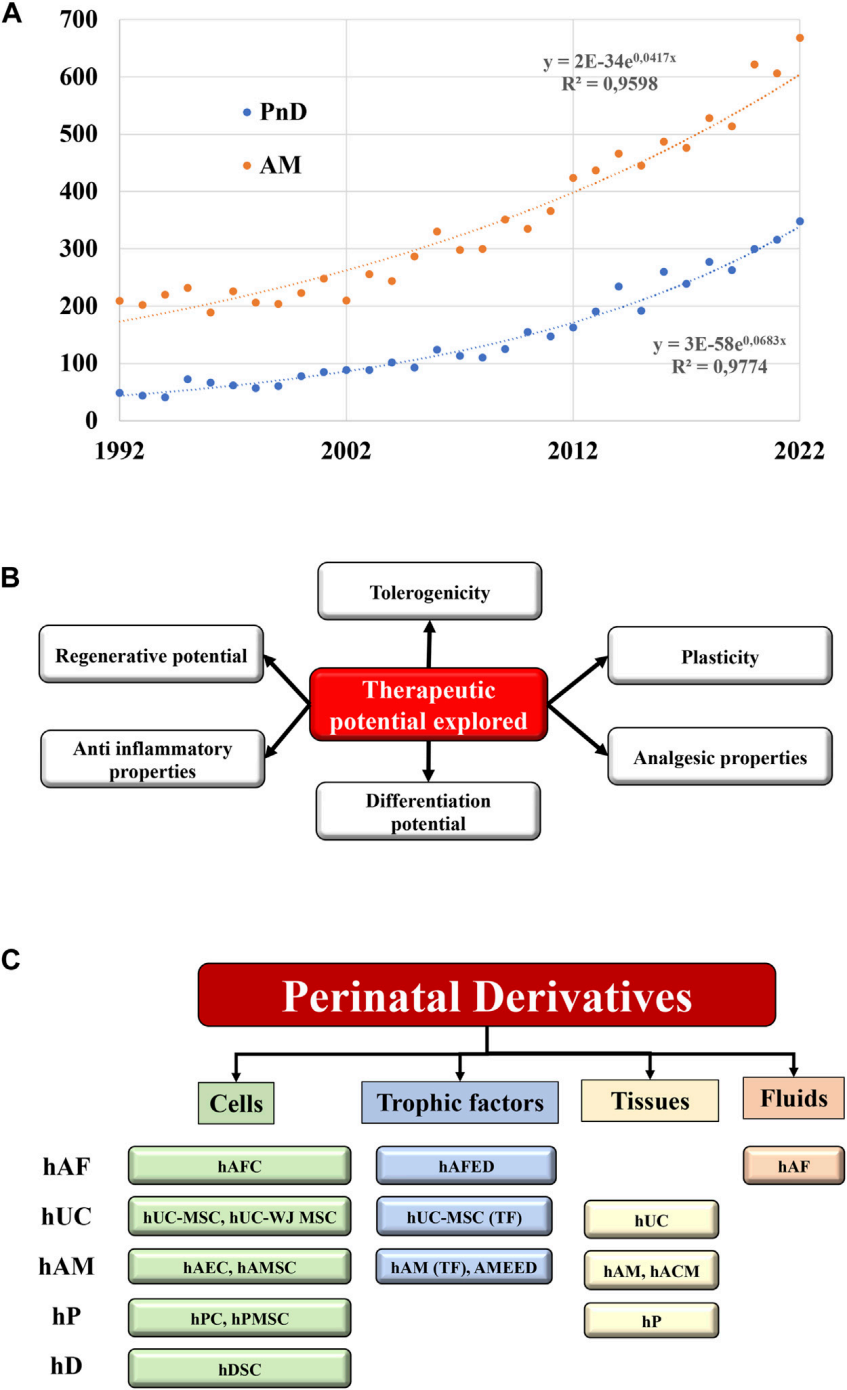
State of the art of the Perinatal derivatives. **(A)** Represents the exponential increase within the last 3 decades of the annual number of references indexed in pubmed.gov site with the keywords “perinatal derivatives” and “amniotic membrane”. The numbers of references doubled every 10 years for the PnD, and every 17 years for the amniotic membranes. **(B)** Therapeutic potential of PnD. **(C)** Different types of PnD utilized in the clinical trials (cells, trophic factors, tissues, fluids) according the different sources of PnD. Abbreviations: hP: human placenta; hUC: human umbilical cord; hAM: human amniotic membrane; hAF: human amniotic fluid; hD: human decidua; TF: trophic factors; hPC: human placental cells; hPMSC: human placental mesenchymal stromal cells; WJ: Wharton’s jelly; MSC: mesenchymal stromal cells; hACM: amnio-chorionic membrane; hAEC: human amniotic epithelial cells; hAMC: human amniotic membrane cells; AMEED: amniotic membrane extract eye drops; hAFC: human amniotic fluid cells; hAFED: human amniotic fluid eye drops; hDSC: human decidua stromal cells.

The scientific community is experiencing a particularly growing interest for the medical use of perinatal products. During the last 3 decades, an exponential amount of reports described the use of human perinatal tissues and cells in a plethora of preclinical settings, supporting their implementation into regenerative and interventional approaches (Figure 1A). This is based on the unique characteristics and features shown by perinatal derivatives largely ascribed to their origin and physiological role, supporting proper foetal development during gestation and providing protection against deleterious maternal immune-recognition (Figure 1B).

Perinatal derivatives (PnD) include fetal and maternal tissues (e.g. human amniotic membrane [hAM]), multipotent stromal cells (MSC) and stem cells embedded in different parts of the placenta or human umbilical cord (hUC). Also, tissue/cellular extracts or secreted mediators such as extracellular vesicles (EVs) represent promising candidates in regenerative medicine, once validated and proved safe and effective in registered clinical trials (Silini et al., 2015; Silini et al., 2017; Torre and Flores, 2020) (Figure 1C).

Among PnD, hAM has a long history of clinical use. For almost one hundred years hAM tissue has been applied in the treatment of skin injuries, in periodontics, orthopedics, the management of female reproductive tract lesions and, particularly, for the assessment of injury on ocular surfaces (Ashworth et al., 1986; Silini et al., 2017; Murri et al., 2018; Sittadjody et al., 2021). Due to the extensive knowledge available on the clinical application of hAM, the commercial use of the matrix is also already established or approved for use by many national authorities.

As the pinnacle of the current state of the art for its more common use both, hAM and hAM extracts, have been successfully shown to promote corneal healing. In a recent development it was demonstrated how the application of hAM along with hAM extract eye drops (AMEED) can synergistically promote stability of the ocular surface and regeneration of corneal epithelium after chemical burns. Notably, this treatment showed to be devoid of undesired effects like causing persistent epithelial defect (conjunctivitis and vascularization of the cornea) or inflammation, conditions that usually appear when resorting to traditional treatments with antibiotics or corticosteroid drops (Murri et al., 2018). The aforementioned combined treatment constitutes a good example on how PnD provide resourceful clinical options for the management of difficult entities in comparison to conventional treatments. Nevertheless, AMEED is only available in Italy, Spain, United States and other markets currently without US-FDA regulation.

Beyond hAM and its extracts, PnD cells are lately receiving particular attention for their broad differentiation potential and tolerogenicity. Among naturally occurring stem cells, PnD are regarded with the greatest potential for cell therapy and regenerative medicine, as cells derived from the placenta do possess unique plasticity and differentiation properties (Bailo et al., 2004; Silini et al., 2020). As per their obtention, only in the last couple of decades new methods of isolation have been tested and improved to purify stem cells derived from different placental regions, human amniotic fluid (hAF), hAM, chorion, hUC (including Wharton’s jelly [WJ]) and cord blood. From these origins, placenta and cord blood have been more intensely examined. The placenta is a known rich source of both stem and stromal cells with demonstrated therapeutic potential in a variety of disease models; being relatively easy to isolate and showing a stable behaviour *in vitro* (Caprnda et al., 2017; Torre and Flores, 2020). Similarly, clinical trial evaluation of safety, efficacy and therapeutic potency of PnD stem cells from other origins in various diseases is increasing.

Contrary to the wide and old routine use of hAM, therapies using cells and EVs have yet to prove their suitability and safety. There has been an increasing diversification of MSC products in the past decade, with preferential use of BM-MSCs until 2008, but increasing diversification since then with now equal use of BM, PT (perinatal tissues), and AT (adipose tissues)-derived cells, with a strong trend for PnD to become the most popular source in the past 2 years. Whereas Meta-analysis has demonstrated that bone marrow MSC infusion is safe (Moll et al., 2019), MSC products from different sources are not investigated enough. With the introduction of the new hemocompatibility characteristic (Moll et al., 2020; Moll et al., 2022) a tool is available with which the safety of the systemic intravascular administration of MSC/EV-products can be examined. Therapies derived from PnD cells and tissues might become advanced therapy medicinal products (ATMP), and EVs biological products through their medical use. The ATMP category englobes products based in recombinant nucleic acids and/or engineered cells and/or tissues. Within ATMP, PnD mainly fall into two sub-lists, somatic cell therapy medicinal products and tissue-engineered products (Iglesias-Lopez et al., 2019). The requirements for these applications are correspondingly much higher, and more clinical investigation via clinical trials is necessary.

Yet, although the evidence of PnD usefulness exists and the interest in the field is obvious, no clear guidelines can be found as how to compare the diversity of PnD origins and applications through clinical trials. Since research in this field is growing such guidelines are essential and defining criteria for clinical use of PnD should be improved. In this study, the current state of PnD and PnD stem cells knowledge in clinical trials was analyzed, in an effort to discriminate different valuable research areas.

A database of clinical trials using PnD tissues, PnD stem cells as well as trophic factors (TF) was created and it is discussed. Moreover, this analysis allowed to create a questionnaire which will be useful to define key clinical trial information significant for the development of PnD products.

### 1.1 Registered clinical trials with perinatal derivatives

By definition, a clinical trial constitutes a research study performed following a clinical setting with the specific intent to evaluate the effects of novel health-related interventions in humans. Such prospective biomedical or behavioral research studies, involving healthy human participants and/or selected groups of patients, are designed to address specific therapeutical issues moving from safety and efficacy to management refinement and market assessment. They are regularly conducted based on previous approval by local ethic committees and health authorities. Such stakeholders are responsible for carefully balancing the risk to benefit ratio of the proposed intervention, and for vetting the initiative when the benefit does not justify the risk. Depending on the product type and development stage, researchers initially enroll volunteers or patients into small pilot research and subsequently conduct progressively larger scale comparative studies. In consequence, clinical trials are commonly subdivided into four different phases: *Phase I* studies, also referred as Early Phase or as “first-in-human” studies if a new substance is tested for the first time, imply testing a new drug into a small group (n = 10–15) of healthy volunteers for safety and administration protocol evaluation; *Phase II* clinical trials aim to evaluate the biological activity or clinical effect of the treatment on a sizable group of patients (n = 50–300), allowing for additional analysis for setting optimal or even tailoring therapeutic conditions according to patients genetic background and metabolic rate to minimise non-desired effects; *Phase III* studies usually consist in a randomized controlled multicenter trial on a large patient cohort (n = 300–3,000 subjects), where efficacy of the new intervention is assessed in comparison to treatments regarded “gold standard”; *Phase IV* constitutes the latest stage and is commonly referred as post-marketing surveillance because involves continuous safety surveillance from official market clearance to detect and confirm any rare or long-term adverse effects over the patient population (n > 1,000–10,000).

As it will be discussed in detail, during the last 10 years numerous clinical trials including PnD for the treatment of a plethora of medical conditions have been registered. Although spanning across all four trial phases, some already finished and many currently ongoing, the bulk of them are Phase I clinical trials.

## 2 Materials and methods

As part of our COST Action (CA17116; https://www.sprint-cost.org/) we created a questionnaire exclusively regarding the use of perinatal cells to have a distinct compilation of the different items required to properly know how a clinical trial has been designed and conducted. At the same time, we realized the lack of important information related to certain data significant to understand and replicate the procedures. Therefore, we inserted into the questionnaire information that should be included as standard into clinical studies when they are reported to the public databases. We have included this document as **Annex 1** with the following sections:

• Perinatal derived product/indication
• Clinical trial information
• Manufacturing (from PubMed and other resources)

This study is focused on analyzing and commenting data obtained from the United States Clinical Trial Registration Portal (clinicaltrials.gov) for 10-year period from 01/01/2011 to 07/10/2020. To successfully gather all PnD-related clinical trials in the database, the following search-terms were used (“cell” OR “tissue” OR “secretome” OR “scaffold” OR “fluid” OR “vesicle”) AND (“placenta” OR “amnion” OR “chorion” OR “umbilical cord” OR “amniotic” OR “decidua” OR “villi”). Cord blood was not taken into account in this study, because of the very wide use made of it as a source of hematopoietic stem cells. Additionally, given the focus of this review in PnD other than cord blood, this word was excluded from the search criteria.

**FIGURE 2:**
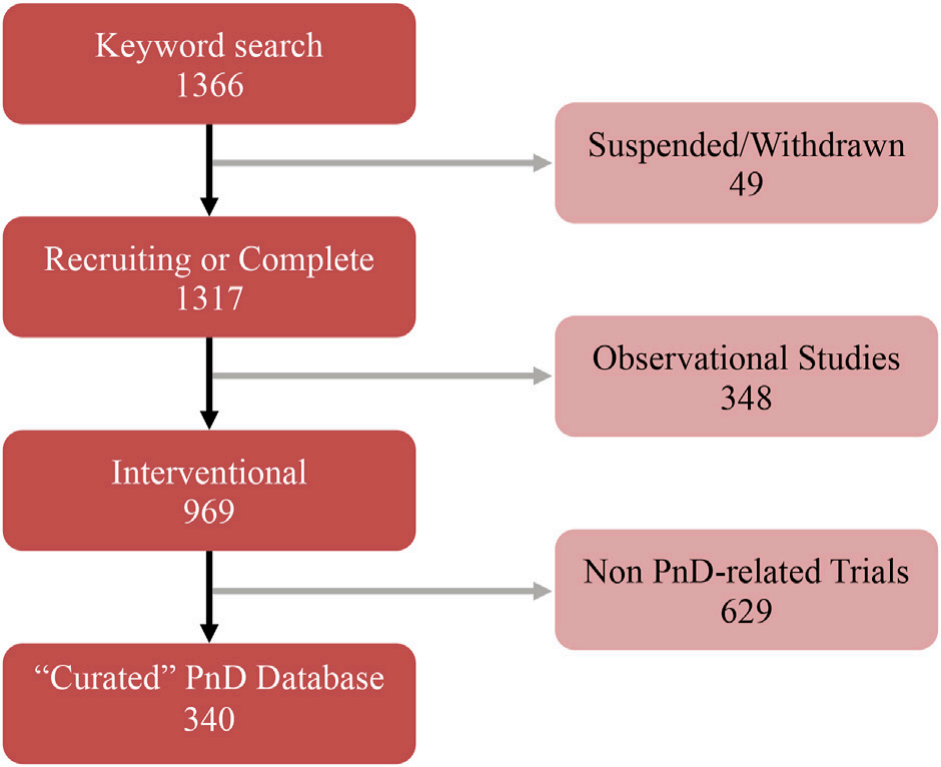
Summary of the several curation steps applied to the keyword search of PnD-related clinical trials.

The search was focused exclusively on interventional clinical trials using human PnD, excluding cases where the studies had been suspended or withdrawn (Figure 2). From the keyword-based search, a total of 1,366 clinical trials were identified. After removing studies with the status of “unavailable”, “no longer available”, “suspended” or “withdrawn”, 1,317 clinical trials remained. Then, all observational studies were also removed from the listing, which led to a list of 969 clinical trials. Given that clinicaltrials.gov included all clinical trials registered within the stipulated time frame, a manual curation step was performed with the aim to classify PnD clinical trials according to the use of either “cell”, “secretome” or “scaffold” forms. For this purpose, 14 reviewers were assessing the different clinical trials (969) to decide which of them were fulfilling the proposed criteria. After this manual curation step, a short-list comprising 340 clinical trials using PnD in an interventional fashion was established (Figure 2; **Annex 2**). For each clinical trial in the database, the following parameters were collected: NCT number, secondary ID, location, status, phase, condition (based on ICD10 classification), gender, age, enrolment, dosage, route of administration, type of therapy (allogenic or autologous) and type of PnD used. Following criteria by Silini et al., 2020, products where some cells were seeded onto a pre-existing tissue (i.e. hAM), biological matrix (i.e. collagen) or into a polymer gel, were defined as “combined” products; when a tissue/material (i.e. bone substitute or others) was combined to a perinatal tissue (i.e*.* hAM), this was considered an “association”. For readers reference, Nucell^®^ represents a good example of combined products as is constituted of hAFC combined with hAM. Inconsistencies in the number of trials analyzed in the evaluation of different parameters arise from the facts that several PnD are investigated in one study and, at the same time, different indications or different nomenclatures were used in the description. In addition, not all trials had information for all considered criteria.

## 3 Results and discussion

### 3.1 Part I: Clinical data base analysis

#### 3.1.1 PnD classification: Categories and origin

Denomination for distinct PnD used in clinical trials within the curated short-list was harmonized applying the proposed consensus nomenclature for human perinatal tissues and cells (Silini et al., 2020). In brief, PnD were classified according to their perinatal origin (human placenta [hP], human umbilical cord [hUC], human amniotic membrane [hAM], human amniotic fluid [hAF] and human decidua [hD]); and according to their final presentation (tissue, cells or TF). Note that TF category regroups conditioned media from cells and/or tissues, and contains also fluid eye drops such as amniotic membrane extract eye drops (AMEED) or human amniotic fluid eye drops (hAFED). Although trophic factors (TF) could have been categorized as secretome, we preferred to stick to the term TF as it was the word found in the clinical trial categories and not secretome. We also considered that keeping TF would facilitate the search of the database to future readers. The harmonized denominations found for PnD in the clinical studies are indexed and classified in Table 1, together with the number of clinical trials testing each PnD.

**TABLE 1:** PnD classification and number of clinical trials according to PnD and their perinatal origin.

Origin	Type	PnD	Nr. clinical trials
hP	Tissue	hP	2
	Cells	hPC	5
		hPMSC	12
	TF	—	—
hUC	Tissue	hUC	11
	Cells	hUC-WJ-MSC	9
		hUC-MSC	200
	TF	hUC-MSC (TF)	3
hAM	Tissue	hACM	18
		hAM	60
	Cells	hAEC	9
		hAMSC	1
	TF	hAMC (TF)	1
		AMEED	2
hAF	Fluid	hAF	13
	Cells	hAFC	7
	TF	hAFED	1
hD	Tissue	—	—
	Cells	hDSC	5
	TF	—	—

Abbreviations: hP: human placenta; hUC: human umbilical cord; hAM: human amniotic membrane; hAF: human amniotic fluid; hD: human decidua; TF: trophic factors; hPC: human placental cells; hPMSC: human placental mesenchymal stromal cells; WJ: Wharton’s jelly; MSC: mesenchymal stromal cells; hACM: amnio-chorionic membrane; hAEC: human amniotic epithelial cells; hAMC: human amniotic membrane cells; AMEED: amniotic membrane extract eye drops; hAFC: human amniotic fluid cells; hAFED: human amniotic fluid eye drops; hDSC: human decidua stromal cells. Note: due to the fact that some trials included more than one PnD the total number of studies is more than the analyzed ones (n = 359 versus 340).

**FIGURE 3:**
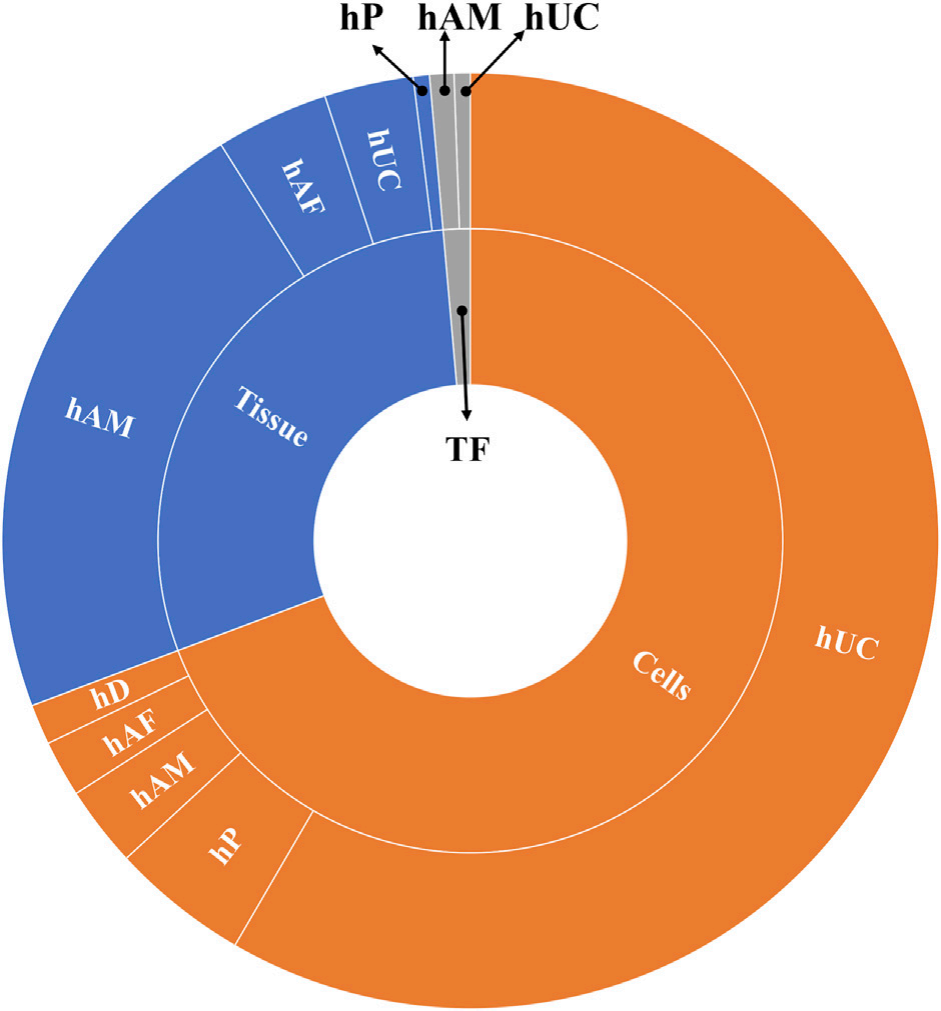
Different uses of cells and tissues in the selected clinical trials analyzed. Abbreviations: hP: human placenta; hUC: human umbilical cord; hAM: human amniotic membrane; hAF: human amniotic fluid; hD: human decidua; TF: trophic factors; hACM: amnio-chorionic membrane.

In general, most clinical trials concentrate on applying hUC-MSC (n = 200) followed by hAM (n = 60), and just the remaining ones are using other multiples sources (n = 80) (Figure 3). Regarding the product presentation, we found again quite homogeneity, as the majority of clinical trials were conducted using cells (n = 248) and/or tissue (n = 104), while just a few (n = 7) involved TF. It is worth noting that all of the clinical trials were conducted using allogenic PnD. However, it should be pointed out that autologous cells from different origins were sometimes combined to PnD (Table 2).

**TABLE 2:** Number of clinical trials with combined cells or scaffolds.

Type of PnD	Combined with	Number of trials
Tissue	Bone substitute	8
	Gel	2
	Glue	1
	Limbal (epithelial) stem cells	4
	BM-MSC	3
	Fibroblasts	1
Cells	Gel	8
	Natural membrane	5
	Tissue	3
	Conduct	1
	Cells from cord blood (CB-MNC, UCB-HSC, USC-MSC)	5
	Cells from bone marrow (BM-MNC, BM-MSC)	2
	AD-MSC	1
	Limbal (epithelial) stem cells	1

Tissue (skin graft, hAM); Bone substitute (demineralized freeze-dried bone and mineralized freeze-dried bone allograft, hydroxyapatite, demineralized [freeze-dried] bone Matrix, bone autograft); Natural membrane (epicardial extra cellular matrix, collagen); Conduct: collagen (NeuroRegen Scaffold™); Gel (injectable collagen/injectable; Collagen Scaffold™, fibrin, PRP, plasma-derived biomaterial, hyaluronic acid). Abbreviations: BM MSC: BM, mesenchymal stromal cells; CB MNC: cord blood mononuclear cells; UCB HSC: UC, blood hematopoietic stem cells; BM MNC: BM, mononuclear cells; UCB MSC: UC, blood MSC; AD-MSC: adipose tissue-derived MSC; PRP: platelet rich plasma.

#### 3.1.2 PnD combined products

According to the above given data, from a total of 46 clinical trials implementing combined products, again, we found applied PnD were primarily composed by hUC-MSC (n = 22) and secondly by hAM (n = 14). Few remaining combined products associated several PnD, including hAM/hAFC (n = 3), hACM (n = 2), hUC-WJ-MSC (n = 2), hPMSC (n = 1), hAEC (n = 1) and AMEED (n = 1).

Interestingly, certain PnD combinations (hAM, hAM + hAFC and hACM) were preferably associated to bone substitutes ([n = 8], Table 2). In other conditions, we found a great diversity of PnD combined to multiple substrates/scaffolds and/or other biological components (gels, natural membranes, tissues, conduct-gels, glue, etc.); or along with additional cells (limbal epithelial cells, other stem/stromal cells, bone marrow [BM]-MSC, fibroblasts, etc.).

### 3.2 Part II: Clinical trials

#### 3.2.1 clinical trial phases

For Phase I, a total of 20 studies are listed ongoing using tissues isolated from different part of the placenta (mainly hAM but also hACM) and hAF, while 177 are the early phase/Ph.1 studies where human PnD cells have been isolated and later infused in (almost exclusive allogeneic) settings. The largest PnD product currently on test is MSC isolated from hUC (commonly defined as hWJ-MSC), used in 151 registered clinical trials. Few early phase studies are ongoing testing cells isolated from hAM (n = 9) or decidua tissue (n = 5), or from a specific or combined section of the placenta (n = 12). In the last decade there has been an increasing number of safety studies for PnD cell-based products, where safety has been the primary target, sometimes involving Phase 2 characteristic analysis as multiple ascending doses, starting from subtherapeutic to high dose of drug. Such preliminary phase is commonly conducted in a single medical center, where the subject can be monitored for tolerability, pharmaco-vigilance/-kinetic/-dynamics of the PnD product. For Phase II, PnD tissue products have been undergoing evaluation in seven registered clinical trials, while 40 studies aimed to evaluated PnD cells (where hUC-MSC represents 90% of the tested PnD products). Phase III trials are expensive, time-consuming and difficult to design. They are usually run by small biotech companies or medical centers and during the last decade only four trials have been registered using hUC-MSC and five studies using other PnD tissue products. For Phase IV, currently just eight hAM or hACM are under final evaluation, while only one bio-pharma technology involving hUC-MSC is included in this phase.

##### 3.2.1.1 Clinical trial classification according to ICD-10 medical fields

Additionally to the technical assessment on PnD characteristics, clinical trials were also analyzed and sorted by the medical field of application, according to the International Statistical Classification of Diseases and Related Health Problems 10th Revision (ICD-10). Thus, all indications were clustered into ICD-10 chapters, each of them corresponding to a specific medical field.

###### 3.2.1.1.1 ICD-10 limitations and shortcomings management

Even though ICD-10 is a thorough and detailed classification intended for delimiting practice according to the causes of disease, discrepancies appeared between ICD-10 classification and common medical terminology, as used in multiple review articles (Silini et al., 2015; Torre and Flores, 2020; Fenelon et al., 2021; Nejad et al., 2021; Elkhenany et al., 2022). Therefore, some adjustments were done to ease the discussion of the results. The first deviation from ICD-10 was made when clinical trials for some of the medical indications (21 clinical trials, 7% of all clinical trials) could not be easily classified solely in one ICD-10 chapter. To avoid duplications, these 21 cases were included into their main, most relevant, ICD-10.

More than half of the clinical trials in this category (57%) belonged to different forms of wound healing fields (and thus discussed in Diseases of the skin and subcutaneous tissue). The second deviation from ICD-10 occurred when it was found that the clinical trials using PnD for COVID-19 were classified into “respiratory system”, despite ICD-10 classification into “special purposes”. This could be a consequence of the ICD-10 being issued in 2019 when COVID-19 was still not a common reality. The first impression was to include it into “Certain infectious and parasitic diseases”, which would clearly be scientifically sound being it a viral disease. However, finally it was decided to keep those in the “respiratory system disease” due to the fact that most COVID-19 patients developed an acute respiratory distress syndrome (ARDS), which constitutes a respiratory condition. In that sense, the vast majority of clinical trials using PnD for the treatment of COVID-19 were not intended for infection mitigation but for improving lung function and prevention of inflammation associated with ARDS. Moreover, despite deviations from ICD-10, we believe that this decision to not include clinical trials for PnD over COVID-19 into the “special purposes” category seems adequate due to the global impact of this disease involving respiratory complications in recent times.

##### 3.2.1.2 Clinical trials distribution according to ICD-10

**FIGURE 4:**
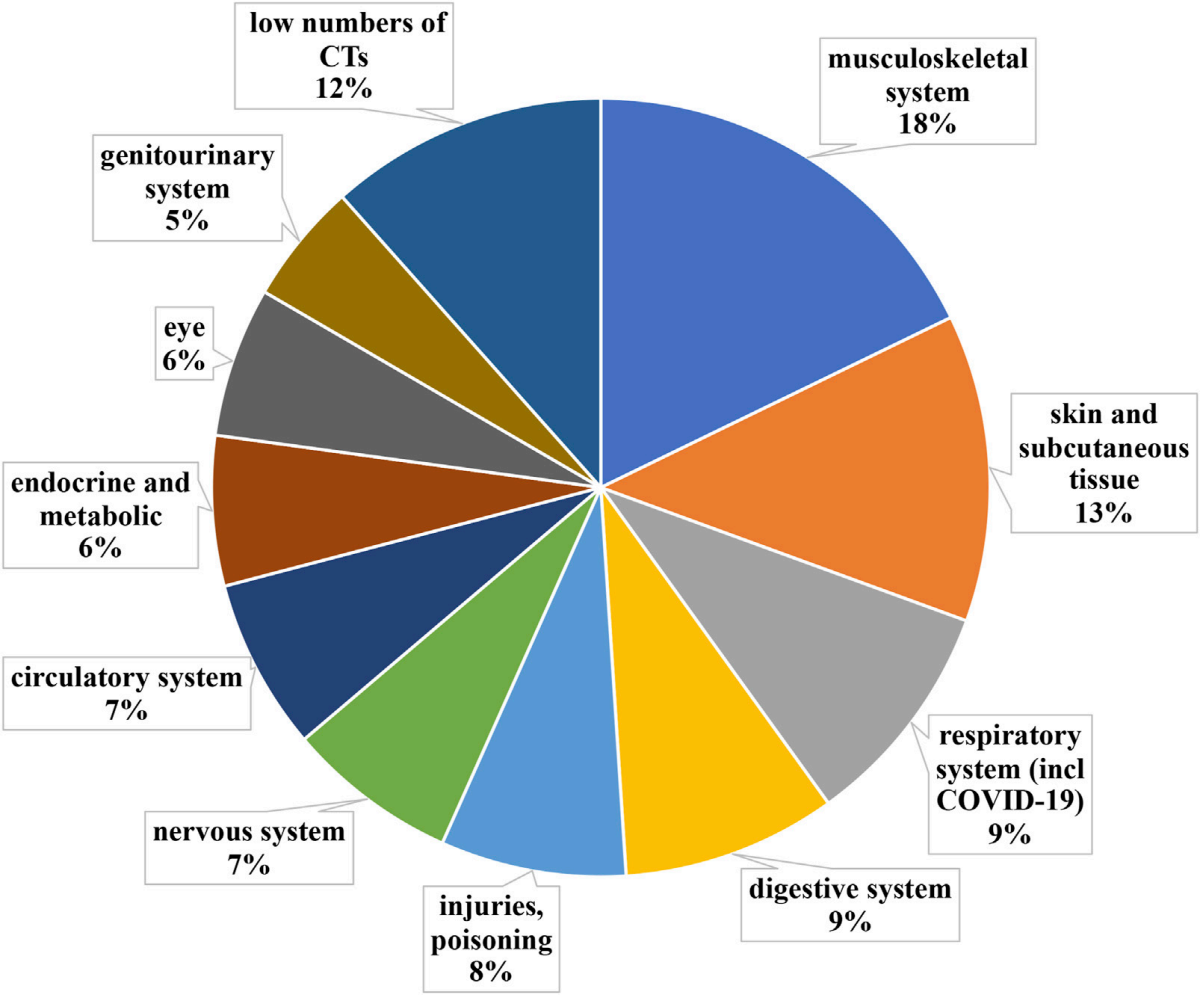
Grouping according to medical fields ICD-10 classification of clinical trials implementing PnD. Medical fields with less than 10 clinical trials were grouped into “medical fields with low number of clinical trials”. The clinical trials using PnD for COVID-19 were classified as a respiratory system disease despite ICD-10 classification into “special purposes”.

The whole 340 short-listed clinical trials were grouped into ICD-10 categories (Figure 4). Medical fields with less than 10 clinical trials were grouped into “medical fields with low number of clinical trials”.

##### 3.2.1.3 Clinical trial activity assessment according to medical fields

###### 3.2.1.3.1 Diseases of the musculoskeletal system and connective tissue

The most active medical field with regards to clinical trials using PnD is Diseases of the musculoskeletal system and connective tissue (ICD-10 chapter XIII), grouping 18% (n = 61) of all short-listed clinical trials. It is worth noting that the majority of clinical trials in chapter XIII fall into two main subdivisions: joint regeneration (n = 23) and rheumatological autoimmune diseases (n = 17).

###### 3.2.1.3.2 Diseases of the skin and subcutaneous tissue

Interestingly enough, in the initial analysis, wound healing and other diseases of the skin and subcutaneous tissue (ICD-10 chapter XII) did not show much activity, even though there are many commercialized products using PnD. The reason for that, as partially explained above, was that as for ICD-10, the wound healing field is mainly present in Chapter XII (n = 20), but is also divided into other chapters, such as IV, XIII, XIV, XVII, XIX (n = 23), altogether representing 13% (n = 43) of all short-listed clinical trials. It is worth highlighting that ICD-10 clearly distinguishes between different causes of wounds (i.e. burns belong to different ICD-10 than diabetic foot ulcers). However, the etiology of the wound does not affect how the wound is treated, as those are usually treated in a similar way independently of the origin. Thus, we have combined all those trials as “diseases of the skin and subcutaneous tissue”. Within this category, the medical indication with the most ongoing clinical trials is diabetic foot ulcers (n = 22). The other significant medical indication is burns (n = 4).

###### 3.2.1.3.3 Diseases of the respiratory system including COVID-19

Diseases of the respiratory system (ICD-10 chapter X + COVID-19) account for 9% (n = 31) of all clinical trials with PnD. In this field, COVID-19 is by far the most addressed condition (n = 18), followed by idiopathic pulmonary fibrosis (n = 2), chronic obstructive pulmonary disease (n = 2) and bronchopleural fistula (n = 2).

###### 3.2.1.3.4 Diseases of the digestive system

This medical field (ICD-10 chapter XI) accounts for 9% (n = 31) of all clinical trials using PnD. Within this category, the far most clinical trials are active for liver cirrhosis (n = 13), followed by periodontal diseases (n = 7) and Crohn’s disease (n = 3).

###### 3.2.1.3.5 Injury, poisoning and certain other consequences of external causes

The next most active medical field was injury, poisoning and certain other consequences of external causes (ICD-10 chapter XIX), grouping 8% (n = 27) of all shortlisted clinical trials. In this case, the medical indications were more diverse, yet, we found two main medical indications being acute Graft-Versus-Host Disease (aGvHD, n = 8) and spinal cord injury (n = 7).

###### 3.2.1.3.6 Diseases of the nervous system

This medical field (ICD-10 chapter VI) accounted for 7% (n = 24) of all shortlisted clinical trials. Inside this field, the main categories were cerebral palsy (n = 5) and Duchenne’s muscular dystrophy (n = 4). One clinical trial was found to be active for each of the next three medical indications: spinocerebellar ataxia, Parkinson’s disease and Alzheimer’s disease.

###### 3.2.1.3.7 Diseases of the circulatory system

Surprisingly, despite being a major mortality cause, diseases of the circulatory system (ICD-10 chapter IX) accounted just for 7% (n = 24) of all shortlisted clinical trials. Within the field, four medical indications had a similar activity for clinical trials: ischaemic stroke (n = 5), myocardial infarction (n = 4), cerebral infarction (n = 4) and cardiomiopathy (n = 3).

###### 3.2.1.3.8 Multiple medical fields

Clinical trials for some of the medical indications (n = 21) could not be classified solely in one ICD-10 chapter and thus were classified “multiple”. However, more than half of the clinical trials in this field (n = 11) fall within the wound healing category, thus will be discussed in diseases of the skin and subcutaneous tissue.

##### 3.2.1.4 Preferred PnD indications according to ICD-10

**FIGURE 5:**
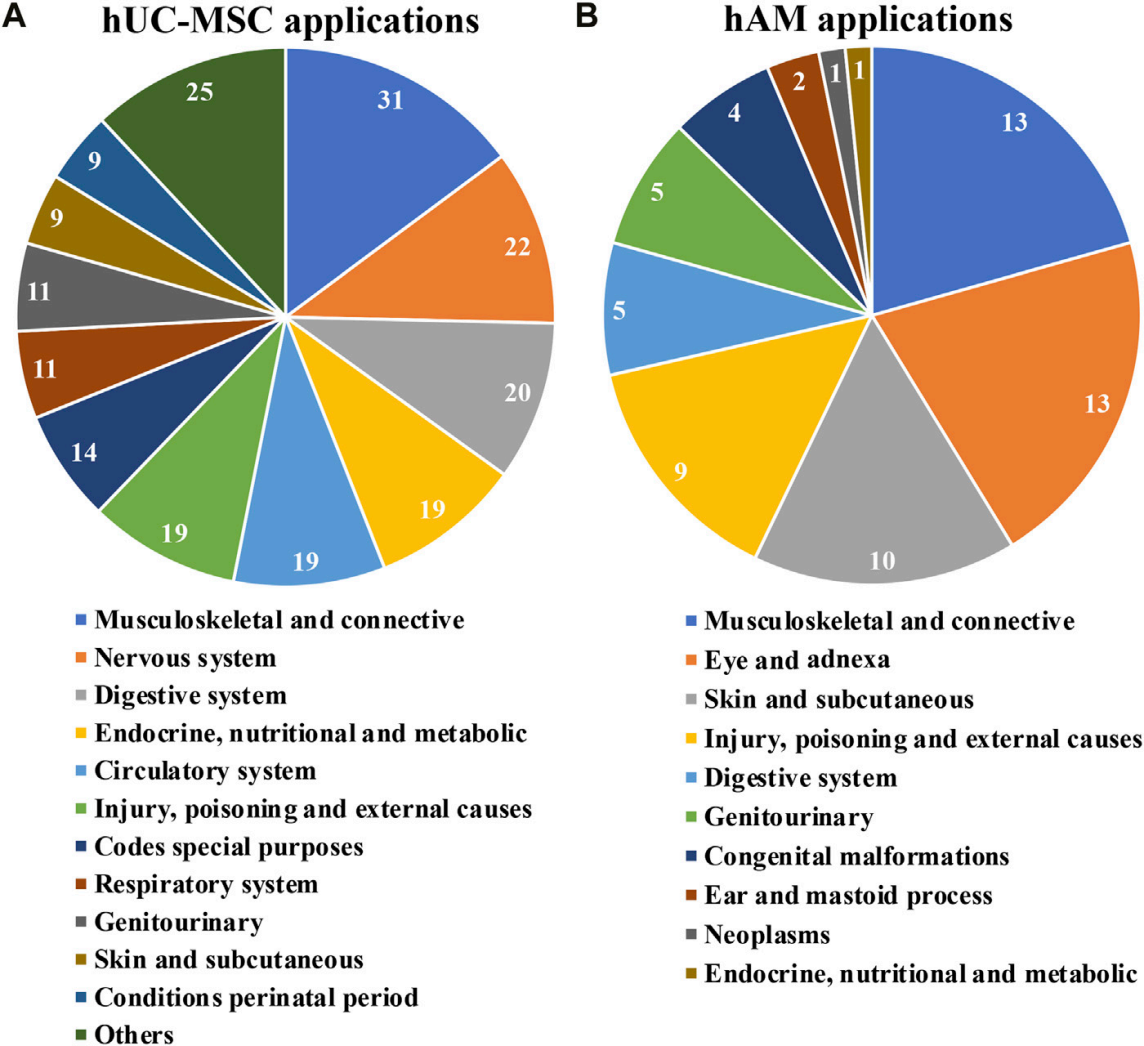
Applications for hUC-MSC **(A)** and for hAM **(B)**; grouping according to medical fields ICD-10 classification of clinical trials implementing hUC-MSC and for hAM respectively.

Additional analysis aimed at understanding which PnD products are prefererably indicated for each medical field identified according to ICD-10 (Table 3). Consistently with other analysis in this work, hUC-MSC and hAM accumulated the bulk of references, 209 and 63 respectively (Table 3; Figures 5A, B). Note: as some studies refer to more than one ICD-10 category, the total number of studies with the corresponding PnD reported here differs from the stated number of studies analyzed with the individual PnD. Also, in a very few studies, two different PnD were used contributing also to the discrepancy. The cases with hAM included two applications with AMEED, and the different trofic factor trials were included in their corresponding cell or tissue of origin categories.

**TABLE 3:** PnD vs. ICD-10.

ICD-10		Cells									Tissue				Fluid	
	General condition description	HUC-MSC	hAMC	hPC	hDSC	hAEC	hPMSC	hUC-WJ-MSC	hAFC	hAMSC	hACM	hAM	hUC	hP	hAF	Total
I	Infectious and parasitic	1	1	—	—	—	—	—	—	—	—	—	—	—	—	2
II	Neoplasms	1	—	1	—	—	—	—	—	—	—	1	1	—	—	4
III	Blood and immune	4	—	1	—	—	—	—	—	—	—	—	—	—	—	5
IV	Endocrine, nutritional and metabolic	19	—	1	—	2	2	1	—	—	1	1	1	—	—	28
V	Mental and behavioural	7	—	—	—	—	—	—	—	—	—	—	—	—	—	7
VI	Nervous system	22	—	—	—	2	—	—	—	—	—	—	—	—	—	24
VII	Eye and adnexa	6	—	—	—	—	—	—	—	—	—	13	—	—	1	21
VIII	Ear and mastoid process	—	—	—	—	—	—	—	—	—	—	2	—	—	—	2
IX	Circulatory system	19	3	2	—	—	1	3	—	—	1		1	—	—	30
X	Respiratory system	11	—	—	—	1	1	1	—	—	—	—	—	—	—	14
XI	Digestive system	20	1	1	—	—	—	—	—	—	4	5	—	—	—	31
XII	Skin and subcutaneous	9	—	—	—	—	1			—	7	10	5		2	34
XIII	Musculoskeletal and connective	31	—	—	—	1	2	1	7	1	3	13	1	1	7	68
XIV	Genitourinary	11	—		1	2	2	—	—	1	—	5	—	—	—	22
XV	Pregnancy, childbirth and puerperium		—	—	—	—	—	—	—	—	—	—	—	—	—	0
XVI	Conditions perinatal period	9	—	—	—	—	—	—	—	—	—	—	—	—	—	9
XVII	Congenital malformations	2	—	—	—	—	—	—	—	—	—	4	1	—	—	7
XVIII	Abnormal clinical	1	—	—	—	—	—	—	—	—	—	—	—	—	—	1
XIX	Injury, poisoning and external causes	19	—	—	4	1	—	1	—	—	4	9	1	—	2	41
XX	External causes of morbidity and mortality	2	—	—	—	—	1	—	—	—	—	—	—	—	—	3
XXI	Factors influencing health status	1	—	—	—	—	—	—	—	—	—	—	—	—	—	1
XXII	Codes special purposes	14	—	—	—	—	2	1	—	—	—	—	—	—	3	20
	TOTAL	209	5	6	5	9	12	9	7	2	20	63	11	1	15	374

Note that number of clinical trials in hUC-MSC, hAMC, hAM, hAF, categories includes TF, AMEED, or hAFED, cases (N = 3, 1, two and one respectively).

For the case of hUC-MSC, clinical trials under assessment span across 21 distinct medical fields (Figure 5A). Interestingly, just nine indications englobed almost 80% of the studies, all of them with at least 11 registered trials so far. This fact would highlight, in a generic manner, the interest of the scientific community in such products. Moreover, “musculoskeletal and connective”, “nervous system” and “digestive system”, had each of them more than 20 trials. Consequently, in these cases, a swift development in the field is to be expected.

Again, a group of only five fields encompassed most of the trials recorded using hAM (Figure 5B). Interestingly, “musculoskeletal and connective tissue”, “skin and subcutaneous tissue” and “injury, poisoning and external causes” show comparable numbers to “eye and adnexa”, probably because the effectiveness of these products recognized for a very long time in these indications has required comparatively fewer clinical trials in the last 10 years. In addition, less expected indications such as “digestive system” and “genito-urinary” had five registered trials, suggesting future progresses in these fields.

#### 3.2.2 Gender consideration

Regarding the gender of patients, clinical trials were mainly conducted on both genders. Nevertheless, there were several cases in clinical trials when PnD had been used only on female because of gynecological indications hAM (n = 5), hUC-MSC (n = 10) and hAEC (n = 4), or only in men because of chronic ischemic cardiomyopathy (hUC-MSC, n = 1 because this gender is more affected than women in these particular condition), prostate cancer (hUC-MSC, n = 1), erectile dysfunction (hUC-MSC, n = 1 and hPMSC, n = 1) or Duchenne muscular dystrophy (hUC-MSC, n = 3).

#### 3.2.3 Age

**FIGURE 6:**
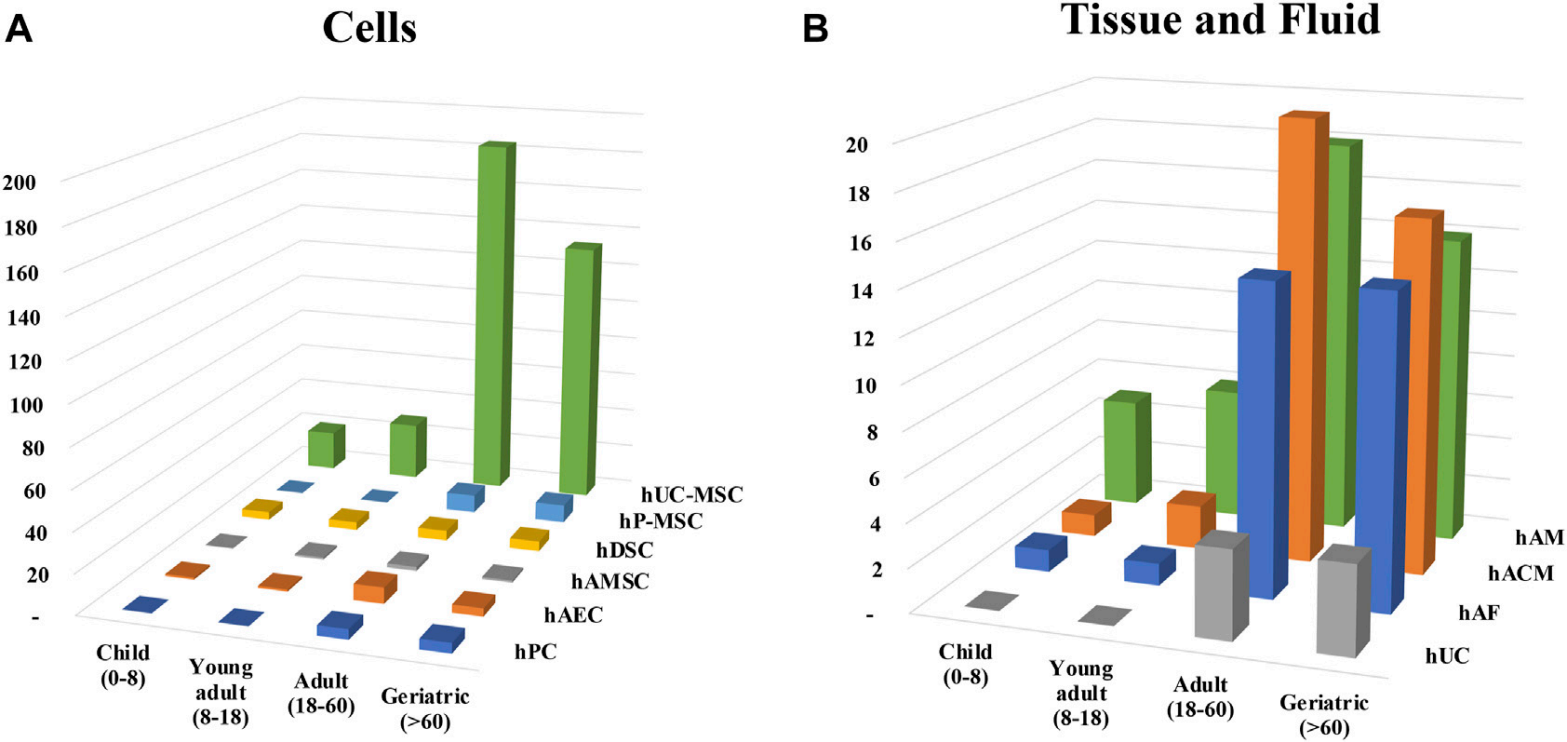
Distribution of clinical trials according to age groups. Clinical trials were classified in **(A)** cells or **(B)** tissue and fluid and the different age group were assigned. Patients were divided and named in four groups depending on the range of age: children (0–8 years), young adults (8–18 years), adult (18–60 years) and geriatric (60 and over).

The evaluation of either the use of cells or tissue in clinical trials clearly show a preferential design of clinical trials for adults or aged people (Figure 6), what is not surprising about regenerative medicine products. Clinical trials specifically designed for children did represent less than 10 percent of the total of analyzed clinical trials. hUC-MSC and hAM were predominant in both children and adults, but specific pathologies were explored in children. As there were only six clinical trials using TF, hAFED or AMEED we did not include them in the analysis. Of these, five were conducted in adults and one in children from 6 months to 3 years old.

#### 3.2.4 PnD manipulation and route of administration

Information available regarding PnD manipulation [according to Annex I to Regulation (EC) No 1394/2007] is often not sufficiently explained in our database clinical trials. Therefore, it is difficult to determine whether the tissue is manipulated or not. Of course, procedures are variable depending on the PnD used. Moreover, for a given tissue the manipulation can differ very much undoubtedly adding an additional variable in the assessment of the therapeutic efficacy on the patient. As an example, this is particularly true for the case of the fetal membranes (hAM, hACM) originating from the placenta which can be decellularized, cryopreserved, devitalized, dehydrated or lyophilized, and sometimes irradiated.

Likewise, the protocols for the preparation of MSC and hDSC are not described, or do not always refer to a precise bibliography. Only the tissue of origin is usually mentioned (placenta, Wharton’s jelly or hUC). Modalities of isolation, expansion and mode of use of cells (fresh or thawed) should be specified in the future. Of note, hAF was always used after filtration and cryopreservation or lyophilization (Table 4).

**TABLE 4:** Distribution of different manipulation and corresponding route of administration among PnD.

Type	PnD	Manipulation	Route of Administration
Cell products (247)	hDSC (5)	Cryopreservation (2)	Sytemic (2)
		n.a. (3)	Sytemic (3)
	hAEC (9)	n.a. (9)	Local (7)
			Sytemic (1)
			Sytemic, Local (1)
	hAFC (7)	Cryopreservation (4) [NuCel®]	Local (4)
		Micronisation and Cryopreservation (3) [ReNu®]	Local (3)
	NK derived from hP (1)	Cell isolation, Expansion (1)	Systemic (1)
	hMSC (224)	Cell isolation (5)	Local (2)
			Systemic (3)
		Cell isolation, Expansion (36)	Local (11)
			Systemic (21)
			Systemic, Local (1)
			n.a. (3)
		Cell isolation, Expansion, Cryopreservation (7)	Systemic (5)
			Local (2)
		Cell isolation, Expansion, Cryopreservation or native (1)	Local (1)
		Cell isolation, Expansion, Selection (1)	Systemic (1)
		Cryopreservation (4)	Systemic (3)
			Local (1)
		Expansion (1)	Systemic (1)
		Cell isolation and Differenciation (1)	Systemic + Local (1)
		Loading on scaffold (3)	Local (3)
		n.a. (165)	Local (56)
			Systemic (96)
			Systemic, Local (2)
			n.a. (11)
Trophic factors/hAF (20)	hAFED (1) AMEED (2) hAMC (TF) (1) hUC-MSC (TF) (3) hAF (13)	Extraction (1)	Local (1)
		Micronization, Dehydration (1)	n.a. (1)
		Cryopreservation (1)	Local (1)
		Decellularisation (1)	Local (1)
		Extraction of exosome particles (1)	Systemic (1)
		Filtration (1)	Systemic (1)
		Lyophilisation (1)	Local (1)
		Purification, Decellularisation (1)	Systemic (1)
		Cell isolation, Expansion (1)	Local (1)
		n.a. (11)	Local (10)
			n.a. (1)
Tissue products (86)	hACM (18) hAM (59)	Micronization and cryopreservation (3)	Local (3)
		Dehydration, Sterilization (1)	Local (1)
		Dehydration (16)	Local (16)
		AM as scaffold for other cells or collagen (3)	Local (3)
		Decellularisation, Dehydration (2)	Local (2)
		Cryopreservation (9)	Local (9)
		Devitalization, Cryopreservation (2)	Local (2)
		Dehydration, Irradiation (1)	Local (1)
		Irradiation (2)	Local (2)
		Lyophilisation and Irradiation (1)	Local (1)
		None (fresh) (2)	Local (2)
		n.a. (35)	Local (35)
	hUC (9)	Cryopreservation (8)	Local (8)
		Devitalization, Cryopreservation (1)	Local (1)

All numbers in brackets indicate the number of clinical studies for that situation.

n.a. = not available.

#### 3.2.5 PnD route of administration and place of application versus ICD-10

For the analysis of PnD routes and frequency of administration we decided to analyze independently the main presentation types.

**FIGURE 7:**
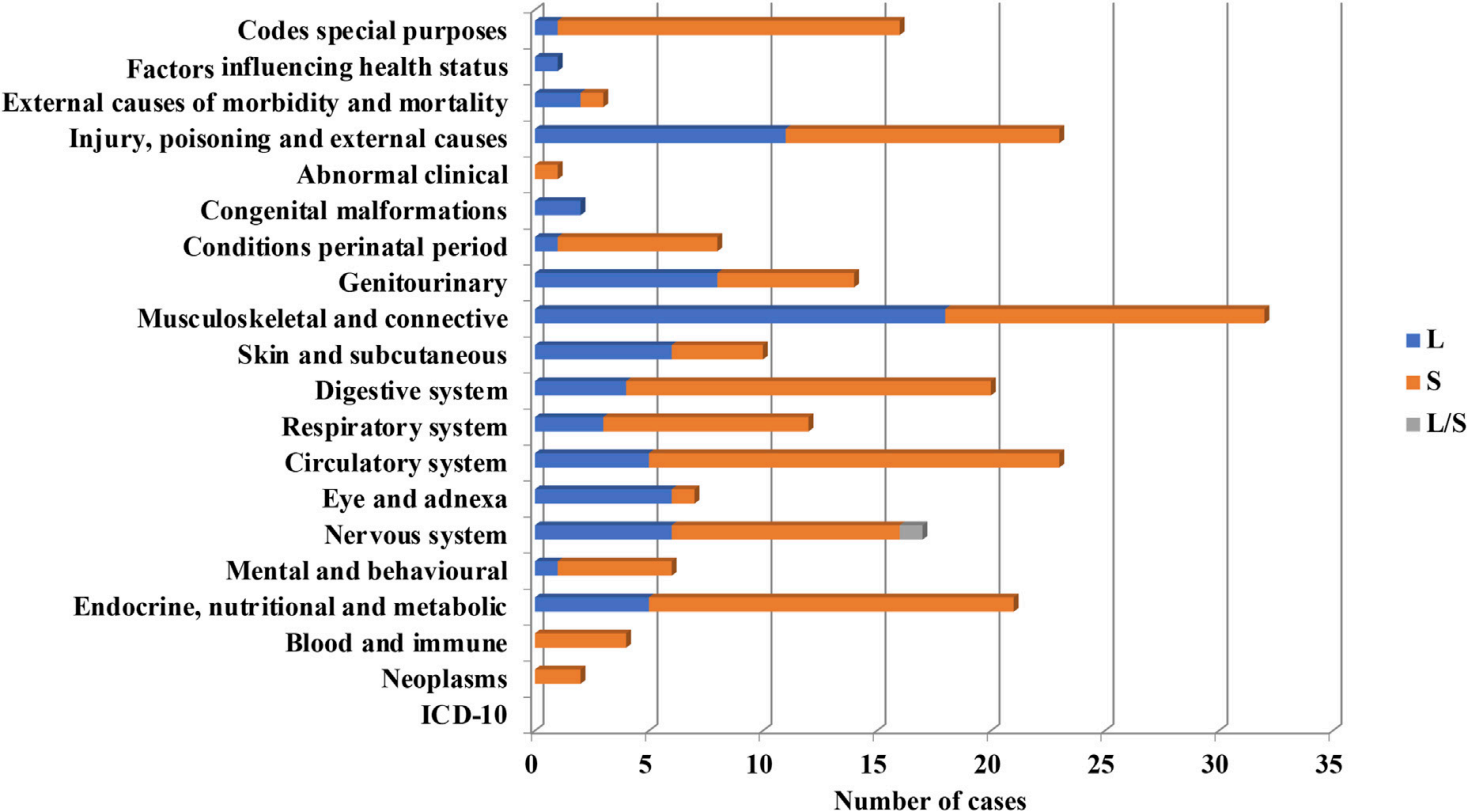
Systemic (S) or local (L) use of PnD cell products for the indication of different ICD-10.

Cell-based products were the most frequently used in clinical trials implementing PnD (n = 248 out of 340, 72.6%). A systemic approach was used in 57.5% cases, while local administration were used in 38.1% and in two clinical trials (0.8%) cells were injected locally and systemically in a simultaneous manner (Figure 7).

From PnD cell products, MSC were the most frequently used (93.5%). Perinatal MSC in most cases were used systemically (60.6%) and locally in the rest of cases (34.2%). Systemic administration of perinatal MSC were used preferentially in the following groups, “Endocrine, nutritional and metabolic diseases”, “Mental and behavioural disorders”, “Diseases of the nervous system”, “Diseases of the circulatory system”, “Diseases of the digestive system”, “Certain conditions originating in the perinatal period” and diseases related to COVID-19 (ARDS, MIS-C) according to ICD-10 classification. In contrast, perinatal MSC were administrated locally more frequently than systemically for the treatment of “Diseases of the eye and adnexa” and “Diseases of the musculoskeletal system and connective tissue” and “Diseases of the genitourinary system” (Figure 7). Note that hAEC were used only in 3.6% of clinical trials utilizing PnD. In contrast to MSC, hAEC-based products were administrated locally except for two out of 10 trials where administration was systemic.

AF-derived cell products were always combined products and included at least hAM and hAF components. They were represented by two commercial products NuCel^®^ and ReNu^®^ (both Organogenesis Inc, United States) which were tested for the treatment of orthopedic diseases (Diseases of the musculoskeletal system and connective tissue). Nucel^®^ was injected into intervertebral disc (n = 3) and for cervical spine fusion (n = 1). On its side, ReNu^®^ was tested for treatment of knee and hip osteo-artritis by intraarticular injection.

Clinical trials implementing hAM also constituted a significant part (22.6%) of clinical trials utilizing PnD. Due to their characteristics, all hAM products are applied locally. The main fields of applications were dermatology, oral surgery and ophthalmology. hAM-based products were most frequently used to treat wounds, burns and ligaments, to avoid scarring after massive surgery and in dentistry and were classified according to ICD-10 as “Diseases of the skin and subcutaneous tissue”, “Diseases of the eye and adnexa”, “Diseases of the musculoskeletal system and connective tissue”, “Injury, poisoning and certain other consequences of external causes” “Diseases of the digestive system” (Supplemental Table S1).

For the case of trials implementing TF, those were mostly administrated locally (n = 16). We found that systemic administration was used just in clinical trials related to ARDS secondary to COVID-19 (Table 5).

**TABLE 5:** Distribution of different routes of administration of TF.

Route of administration	Nr of clinical trials	Percent
Local	16	76.2
Systemic	3	14.3
N.a.	2	9.5
Total	21	100.0

#### 3.2.6 PnD dose and frequency of treatment according to ICD-10

For this section, a subset of clinical trials was analyzed based on the use of PnD MSC, including: hDSC, hUC-WJ-MSC, hAMSC, hPMSC and hUC-MSC. This criterium was favoured in order to ascertain appropriate comparison regarding use in several conditions at different dosages and frequencies. Out of the shortlisted clinical trials, 248 used cells of any nature. Among them, trials using MSC of the described nature amounted up to 225.

Having an internal reference for the proper assessment of regenerative results when comparing patient to patient, independently of health provider or the nature of disease, remains crucial. So, to serve this purpose, we selected trials that were referred to the total body weight (BW) of the patient (kg). Of the 225 clinical trials involving MSC, only 83 had been using mass body as a reference. For the rest of the clinical trials, there was no relative reference, and only three had a surface of the lesion reference. Besides, for 19 of the trials we could refer to a given volume of treatment but without further reference on the BW. Worth noting, just in two trials the reference for treatment performance was assigned to the volume or surface of the lesion (keloids and cartilage defects respectively).

Surprisingly, an appreciable number of clinical trials did not indicate the cell dose or numbers (n = 80), although they reflected the administration frequency (n = 35). This fact increases uncertainty and inconsistency for treatment developments. Yet, when trials were reporting the dosage of the treatment, the frequency of treatment was also given in most cases (90%).

We tried to combine the data of frequency and dosage in our analysis. Because some trials were using different doses, then they were considered independent trials in relation to frequency. When different number of frequencies were considered in a single clinical trial, the highest frequency was used for calculations. In order to understand better, and be able to compare between clinical trials, we established four dose ranges: below one million, between 1 and 10 millions, 10 to 100 millions, or above 100 million cells per kg BW (Table 6).

**TABLE 6:** Dose of PnD MSC and frequency of treatment used in clinical trials referring to BW of the patient.

		Frequency					
		1	2	3	4	5	>5	TOTAL
Dose	<1M	4.4	0.0	5.6	4.4	0.0	3.3	17.8
	1M-10M	25.6	13.3	15.6	11.1	2.2	6.7	74.4
	10M-100M	2.2	0.0	1.1	1.1	0.0	1.1	5.6
	>100M	0.0	0.0	1.1	1.1	0.0	0.0	2.2
	TOTAL	32.2	13.3	23.3	17.8	2.2	11.1	100.0

Our analysis showed that the most frequent treatment was an unique dose (frequency 1) of 1–10 million cells/kg BW (25% of trials) which in almost in half of the cases was aimed to treat pulmonary-related conditions. Forty per cent of the remaining trials used the same cell dose applied two, three or four times. The number of cases for the other doses was so low that we could not have any clear conclusion about the preferred usage of frequencies at those doses.

##### 3.2.6.1 PnD cells administration dose according to ICD-10

Understanding whether the treatment of some human conditions prefer a given dose of PnD cells compared to others might be valuable for future research. To ascertain this, we analyzed the dose use for treatment of the different groups of human conditions. To have a comparable measure, following the previously mentioned criteria, we compared doses referred to the total BW of enrolled patients (Table 7).

**TABLE 7:** Use of cell dose in different groups of medical conditions. The number of cells are referred to kg of BW of the patient.

ICD-10	ICD-10 (General name of condition)	<1M	1M-10M	10M-100M	>10M
I	Infectious and parasitic	—	—	—	—
II	Neoplasms	—	—	—	—
III	Blood and immune	2	3	1	—
IV	Endocrine, nutritional and metabolic	1	7	—	—
V	Mental and behavioural	—	2	—	—
VI	Nervous system	2	1	—	1
VII	Eye and adnexa	—	—	—	—
VIII	Ear and mastoid process	—	—	—	—
IX	Circulatory system	1	5	1	—
X	Respiratory system	—	5	—	—
XI	Digestive system	3	8	1	—
XII	Skin and subcutaneous	—	4	—	—
XIII	Musculoskeletal and connective	1	8	—	—
XIV	Genitourinary	—	2	—	—
XV	Pregnancy, childbirth and puerperium	—	—	—	—
XVI	Conditions perinatal period	—	7	2	—
XVII	Congenital malformations	—	1	—	—
XVIII	Abnormal clinical	—	1	—	—
XIX	Injury, poisoning and external causes	2	12	—	—
XX	External causes of morbidity and mortality	—	2	—	—
XXI	Factores influencing health status	—	1	—	—
XXII	Codes special purposes	3	7	1	1
	TOTAL CASES	15	76	6	2

From the clinical trials providing information about cell doses, the analysis shows how all conditions had a preference for using cells in a range of 1–10 million/kg, (Table 6). Most of the clinical trials analyzed in this group used systemic delivery. Interestingly, certain condition groups were related to treatments below one million cells, amounting for more than 10% of the trials in that category (ICD-10 groups: III, IV, VI, IX, XI, XIX and XXII). Conditions using cells above 10 million/kg BW were ICD-10 groups: III, IX, XI, XVI and XXII. Half of the human conditions groups were using cells only ranging 1 to 10 million cells per kg of BW. The analysis of data from clinical trials that only offered the absolute number of cells (not related to BW) showed a similar order of magnitude per treatment (Supplemental Table S2). Unfortunately, further analysis on absolute amounts and the non-referred units was unpracticable, as in most of the trials the information about doses was not provided.

##### 3.2.6.2 PnD cell dose according to route of administration

**FIGURE 8:**
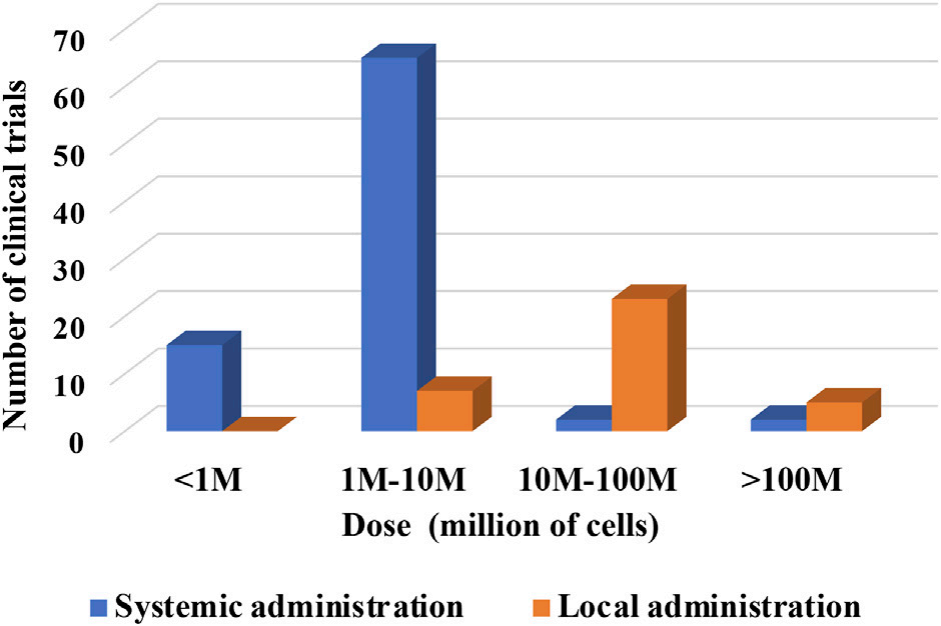
Dose of cells per kg of BW versus route of administration of cells (hPMSC, hUC-MSC, hAMSC).

Understanding limitations imposed by the administration route might also be valuable for future research design (Figure 8). The total number of clinical trials using “systemic” administration was 49, while the total number of clinical trials using “local” was 22.

The analysis indicates a preferential usage of the medium cell doses up to 10^7^ for systemic administrations. In local administrations, the higher doses used were up to 10^8^ cells. Of note, doses in systemic administrations were presented per kg BW. Clinical trials with local administrations did not indicate, with few exception, whether the dosage is designated per kg BW or total infused cells. Yet, it seems fair to assume that most of the local indications are referring to total cell number delivered locally regardless of patient’s BW.

In the case of local administration, just five registered clinical trials indicated the dosage per kg of BW. In 4 cases, a medium cell dose up to 10^7^ cells per kg BW was used. In 2 cases, higher doses were specified (one study used medium and high doses). This would confirm a trend for higher local doses when comparing to systemic administrations indicated per kg BW.

Considering that most clinical trials were focused on adult patients, the total number of cells infused systemically would be similar when comparing to local administrations. Exceptionally, in the clinical study NCT03645525, on the pediatric condition bronchopulmonary dysplasia, up to 20 million cells were instilled per kg BW directly into lungs of extreme preterm new-born children with an average body-weight of 1 kg.

#### 3.2.7 Enrollment

Enrollment represents the number of participants in a clinical study. However, in “clinicaltrials.gov” website, this information is not always available. Indeed, for most of the studies registered, the only information available usually indicated the “estimated enrollment”, i.e. the target number of participants that the researchers needed for the study. Although estimated enrollment is always present, the information regarding the effective number of participants involved was rarely existent and the “recruitment status” being most of the time not updated (“unknown”). Therefore, no conclusion can be made about the enrollment for these clinical studies. However, it should be highlighted that updates should be made, for instance every 6 months, stating the actual number of participants and the recruitment status (few studies stated the “actual enrollment”).

#### 3.2.8 To-be-commercialized products in clinical trials

80 of the 340 clinical interventional trials were conducted on already available commercial products or when a market approval seems to be at least aimed for (to-be-commercialized). From those, 30 clinical trials have used cell products (hUC-MSC, hPC, hPMSC, hUC-WJ-MSC) that already have a registered name, however as far as we know none of them has been approved by any official body to date. In parallel, 48 studies have applied tissue products, mainly as a matrix, and exclusively corresponded to preparation of hAM, hACM or chorion membrane, all commercially obtainable. hAF has been tested in only two studies. Finally, in 14 clinical trials, cells and matrix were used in combination. Of these 78 trials, 52 were registered in the United States, the country where the majority of the manufacturers of these products are located. In Supplemental Table S3-S5, the different commercialized products are listed.

#### 3.2.9 Geographic location of clinical trials

**FIGURE 9:**
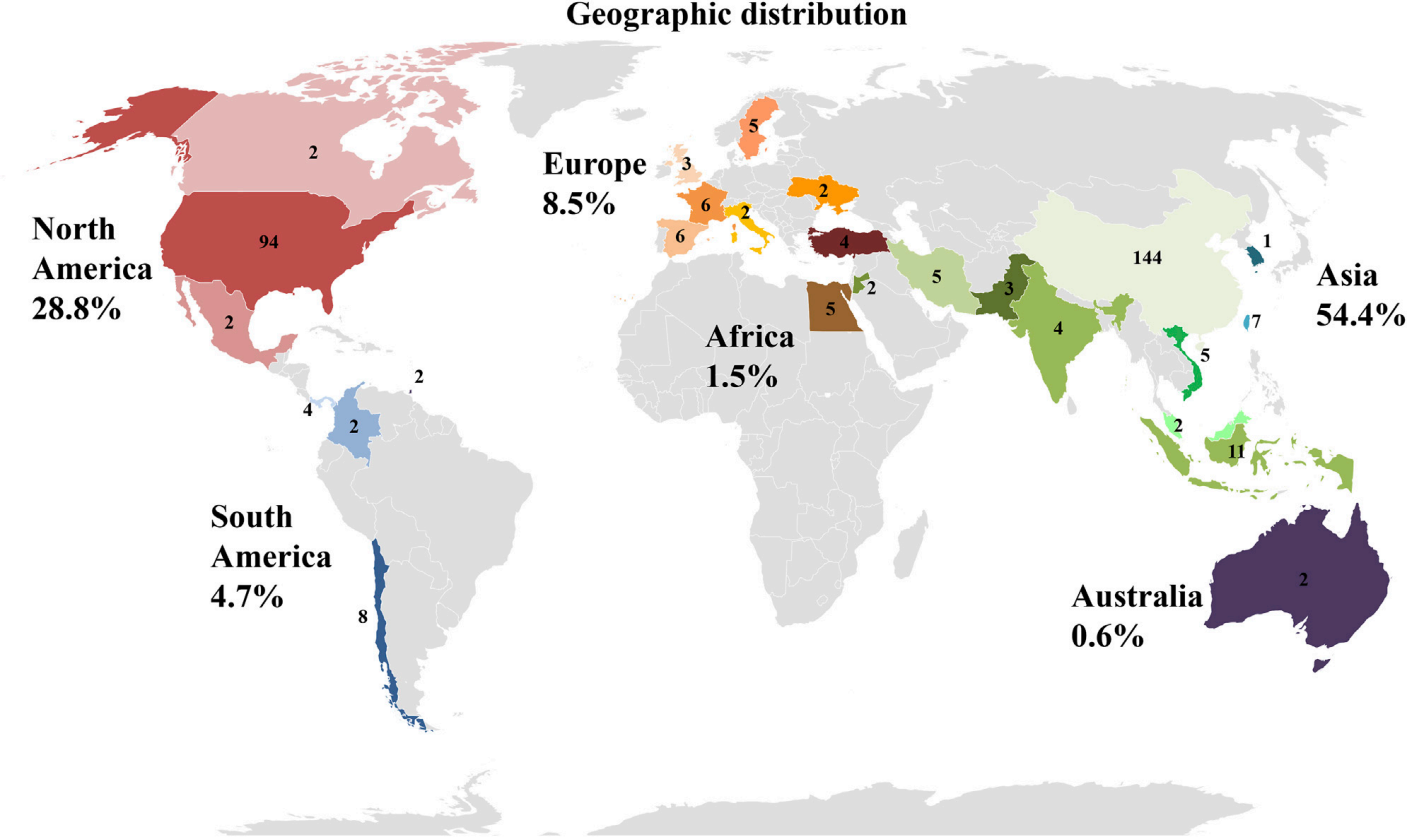
Location of clinical trials with PnD. The number of studies per country is indicated. In addition, the individual countries are grouped into geographical regions. Red colors stand for North America, blue for South America, orange for Europe, brown for Africa, green for Asia and purple for Australia. The number indicates percentage of clinical trials with PnD on the different continents in the period under review.

Data shown in Figure 9, give an overview of the distribution of clinical trials conducted or registered and implementing PnD. It is striking that of the 340 identified studies, 144 are located in China and thus a total of more than 50% of all clinical studies in Asia. With 94 studies in the United States, almost another third are conducted in North America. Surprisingly, only 8% of the studies registered are in Europe. While the majority of the indicated studies in China are designed with cells (MSC of various origins), research in the United States is conducted in roughly equal parts on cells, tissue as matrix, extracts and combined PnD.

Our research has shown that interest in the clinical use of PnD is steadily increasing. With this development, the question of regulation of donation, production and distribution is becoming more important at the same time. Among the information available, no details were found on regulatory aspects related to the clinical trials. When using PnD in the form of the tissues such as hAM, hACM, chorion membrane or hUC, which serve as a matrix in clinical therapy, several commercially available products can already be found. The preparation of these tissues has been in common use for decades and is therefore also accepted by national authorities for use. Further preparations may also be authorized for distribution relatively easily from national authorities in the individual countries, if the use does not take place only under local supervision anyway.

However, the newer, promising therapeutic options with the diverse cells isolated from birth-associated tissues, require much more extensive regulatory measures because they will probably be predominantly classified as ATMP. In Europe, these therapies will only find their way into the clinic if they have first been evaluated and approved via the European Medicines Agency (EMA) through the EU centralized procedure (Flory and Reinhardt, 2013; Celis et al., 2015; Salmikangas et al., 2015; Goula et al., 2020; Nejad et al., 2021).

## 4 Conclusion

Here we proposed to depict the clinical trials using PnD registered on the clinical trial website (https://www.clinicaltrials.gov). To our knowledge, this is the first article that precisely describes these trials over a period of 10 years. It allows to have a synthetic view on the efforts of the scientific community to evaluate the therapeutic potential of these products, although it should be noted that of course we do not have to date exhaustive and reliable data concerning their actual efficacy in every trial. Consequently, our intention is not to endorse any efficacy of the proposed treatments, but to throw light on the attempts to evaluate these therapies in different indications.

Nevertheless, PnD have already demonstrated their suitability for promising therapies. Our results indicate that the PnD that have been tested are very varied and are represented by cells, tissues or TF (including the amniotic fluid itself). None were a gene therapy product, but one can imagine that in the future cellular or tissue PnD could also constitute raw materials easily available and usable to prepare genetically modified products. The indications are also very diverse, although some have been explored more often (e.g. musculoskeletal and connective tissues). Due to the diversity of indications, we have chosen to group them according to ICD-10, even if this classification has its limits and includes pathologies that can be very different.

PnD tested in the clinical trials displayed striking differences in their relative frequencies; one can easily speculate that it is due to different “product life cycles“. For instance, in accordance with the fact that hAM was the first PnD to show their therapeutic efficacy in regenerative medicine, we can still find them very represented in the clinical trials recorded over the period considered. This undoubtedly reflects the need to extend the use of hAM to indications other than those accepted until now by the national regulatory authorities, most often limited to ophthalmology, and this despite an abundant literature on these applications. As proof, the eye and its adnexa represented only 20% of the indications tested in the clinical trials for hAM. The extension of these indications can only be encouraged if these protocols confirm therapeutic efficacy, because the absence of ATMP status for hAM allows the easy preparation and availability of these products. At the same time, the issue of the cost and the financial coverage of these new therapeutic approaches should also be facilitated by the national authorities, so as to make them truly accessible to patients.

Interestingly, hUC-MSC actually represented the first product tested (more than half the trials). This reflects the enormous hope that MSC have held due to their multipotency and their relative ease of preparation, and despite their heavier regulatory status as ATMP. In fact, these cells have played a large part in the recent development of regenerative medicine. Since then, issues about ploidy and their thrombotic potential have nevertheless weighed this enthusiasm. In particular for the systemic intravascular administration of MSC/EV-products, the expression of highly procoagulant tissue factor (TF/CD142) and hemocompatibility aspects are of crucial relevance for their safety profiles in patients, which is of particular importance for some PnD products (e.g. DSCs) due to their intrinsically high expression of TF/CD142 in the placenta, to counteract bleeding. This is important for the appropriate mode of delivery (e.g. systemic infusion vs tissue injection or ectopic use) (Moll et al., 2015; Ringden et al., 2022). One can imagine that the organization of these clinical trials in very diverse indications was necessary to define the pathologies likely to respond to these treatments.

Concordantly, combined products used mainly these flagship products, in combination to multiple substrates/scaffolds and/or other biological components/cells, including possibly another PnD. Indeed, hAM was more frequently associated to bone substitute for needs of the condition (orthopedic, maxillo-facial and oral surgeries). On the contrary, PnD cells were combined with gels to facilitate their local administration. However, the majority of the PnD were used alone. While tissue engineering has shown a wide interest and is largely developed in experimental research from PnD, in clinic, hAM was poorly used as scaffold for cells seeding (n = 8); PnD cells slightly more exploited (n = 17). This combination of products seems profitable for future marketing.

All of the other PnD, which are diverse, accounted for only 23% of clinical trials. They can be considered as “emerging” products, which will certainly require more time to prove their effectiveness compared to standard treatments and to reach the patient’s bedside.

Moreover, most of PnD were tested in Phase I clinical trials; it echoes the very great diversity of products and indications, and shows that the investigation of the therapeutic potential of PnD is carried out in all directions and still is in an initial pioneering phase, even if some commercial products already exist, in particular in the United States. Therefore, while some protocols will not show positive results, some others may be the source of marketed products constituting a breakthrough for certain indications.

**FIGURE 10:**
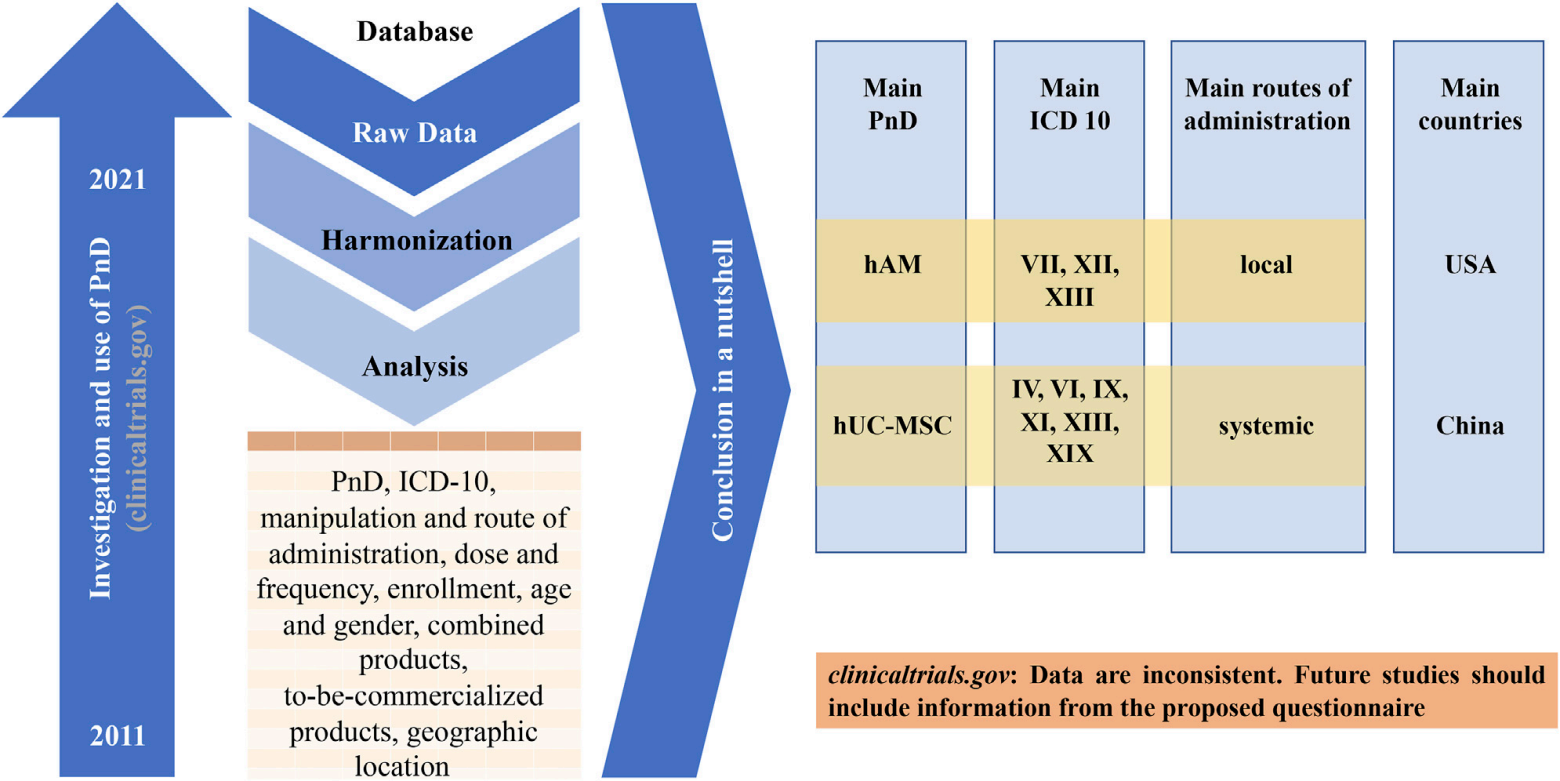
Graphical abstract of the study. The United States Clinical Trial Registration Portal (clinicaltrials.gov) was searched for 10-year period from 01/01/2011 to 07/10/2020 with different search-terms. From the keyword-based search, a number of clinical trials were identified. After removing studies for curing, the remaining 340 trials were analyzed. The main conclusions are shown and a more detailed registry is proposed.

During this study, we observed that the clinical trial website (https://www.clinicaltrials.gov) is mainly built for administrative purpose. Researchers and investigators have difficulties to find relevant informations. There is no uniform specifications as to what informations must be available for the entry. For example, the final number of included patients, the dose used and the type of manipulation of the PnD are often missing. Furthermore, there is usually no conclusive assessment even for studies that have been completed for some time and Publication DOI are very often absent. Frequently, a product name is used suggesting a market approval even if the PnD is neither yet approved nor commercially available. Conversely, it is not always stated if the PnD is already available for purchase. Regulatory aspects are also not addressed. However, all this information would be important in order to be able to assess the success of a study and thus enable the way into clinical practice. Therefore, the authors recommend that all informations from the questionnaire created in this work should also be made available in the future when entering it into the database of the clinical trial website (Figure 10).

Finally, in order to guarantee the safety and quality of these upcoming products for the recipients, further requirements will arise in the future. This concerns all relevant areas from tissue donation to processing and distribution. The development of processing protocols and the establishment of storage facilities in cell and tissue banks are necessary steps on the way to the new therapies. It will be essential to develop standards for this that also comply with legal requirements. From this point of view, it is striking to note that around 70% of the clinical trials were launched by China and the United States; therefore European Countries, Canada and India have lagged considerably behind in the evaluation of these products. In this context, it would be desirable if the regulation on clinical use and especially on commercial use were harmonized. Since the majority of studies with cells have so far been conducted in China, while the largest number of studies with hAM have been conducted in the United States, this will be a particular challenge.

## Data Availability

The original contributions presented in the study are included in the article/Supplementary Material, further inquiries can be directed to the corresponding authors.
