# Supplementary material for: Perinatal derivatives application: Identifying possibilities for clinical use
Source: Front Bioeng Biotechnol. 2022 Oct 11;10:977590. doi: 10.3389/fbioe.2022.977590 (PMC9595339; doi:10.3389/fbioe.2022.977590)
Supplement: Supplementary file 1 [file DataSheet1.zip › Supplementary tables and annexes/Annex 1.pdf]

Clinical trial information for perinatal derived products, registration date.

Perinatal derived product/indication

1. Source of information
   1.1. Clinicaltrials.gov
   1.2. Pubmed.com
   1.3. Local health regulatory authority
   1.4. Others

2. Which component of the product has a perinatal origin?
   2.1. Cells
   2.2. Tissue
   2.3. Secretome
   2.4. Scaffold
   2.5. Fluid

3. Clinical trial ID

4. Publication DOI

5. Indications (to be defined by other groups)

6. Type of therapy
   6.1. Monotherapy
   6.2. Combined therapy

7. Type of application
   7.1. Autologous
   7.2. Allogeneic

8. Perinatal derivative
    8.1. Placenta
    8.2. Amnion
    8.3. Chorion
    8.4. Umbilical cord
    8.5. Amniotic fluid
    8.6. Decidua
    8.7. Villi
    8.8. Other (EVs?)

9. If any perinatal cells used, please specify (Refer to WG1 classification, pending!)

10. If any non-perinatal-derived product used, please specify.

Clinical trial information

11. Clinical Trial Location

12. Phase of the clinical trial

13. Current status of the clinical trial.

14. Clinical trial sponsor

15. Product name
    15.1. Stage of clinical development
    15.2. Availability to patients
    15.3. Commercialization status (if available)

16. Route of administration

17. Cell Dosage/Concentration/Volume

18. Target enrollment number

19. Patient age category (pediatric, adult and/or senior)

Manufacturing (from PubMed and other resources)

20. Regulatory pathway
    20.1. Medical device regulation[1]
    20.2. Human cell and tissue regulation[2]
    20.3. Advanced therapy medicinal product (ATMP)[3]
    20.4. Other different from EU regulations (specify)[4]

21. How is this product stored before administration?
    21.1. Frozen
        21.1.1. Temperature (please indicate)
        21.1.2. Cryopreservation medium (if applicable)
    21.2. Room Temperature
    21.3. 4 °C

22. Product vehicle administration? (please specify)

23. Which steps did the product undergo
   23.1. Minimally manipulated product (cutting, grinding, shaping, centrifugation, soaking in antibiotic or antimicrobial solutions, sterilization, irradiation, cell separation, concentration or purification, filtering, lyophilization, freezing, cryopreservation, vitrification)[3]
   23.2. Substantially manipulated product[3]
      23.2.1. Expansion (either in vitro or bioreactor)
      23.2.2. Gene modification
      23.2.3. Other steps (please specify)
   23.3. Other different from EU regulations (specify)[4]

24. Culture medium formulation
   24.1. Xenogenic serum
   24.2. Medium without xenogenic serum
      24.2.1. Human platelet lysate
      24.2.2. Human serum
      24.2.3. Chemically defined
   24.3. Other supplementation
   24.4. Serum-free

25. Product quality control
   25.1. Sterility (including Mycoplasma)
   25.2. Cell viability
   25.3. Morphology

25.4. Phenotypic studies (specify)
25.5. Immunophenotype (specify)
25.6. Functional assays (identify)
25.7. Tumorigenicity
25.8. Genetic stability
25.9. Immunogenicity
25.10. Endotoxin

26. Pre-administration step (if necessary)
    26.1. Washing
    26.2. Reformulation (please specify)

1. DIRECTIVE 2001/83/EC OF THE EUROPEAN PARLIAMENT AND OF THE COUNCIL of 6 November 2001 on the Community code relating to medicinal products for human use

2. REGULATION (EU) 2017/745 OF THE EUROPEAN PARLIAMENT AND OF THE COUNCIL of 5 April 2017 on medical devices, amending Directive 2001/83/EC, Regulation (EC) No 178/2002 and Regulation (EC) No 1223/2009 and repealing Council Directives 90/385/EEC and 93/42/EEC

3. REGULATION (EC) No 1394/2007 OF THE EUROPEAN PARLIAMENT AND OF THE COUNCIL of 13 November 2007 on advanced therapy medicinal products and amending Directive 2001/83/EC and Regulation (EC) No 726/2004

4. FDA regulations, or other countries regulation.
