# Supplementary material for: Perinatal derivatives application: Identifying possibilities for clinical use
Source: Front Bioeng Biotechnol. 2022 Oct 11;10:977590. doi: 10.3389/fbioe.2022.977590 (PMC9595339; doi:10.3389/fbioe.2022.977590)
Supplement: Supplementary file 1 [file DataSheet1.zip › Supplementary tables and annexes/supplemental table 4.pdf]

Supplemental table 4. Different commercialised or to-be-commercialised matrix-based products found in the database.

Matrix	Active substance	Company	Phase	No. of trials
ActiveBarrier®	hAM	Skye biologics	na	1
AlloWrap	hAM	Allosource	na	1
hAM Iran tissue product	hAM	not stated	na	1
AmnioExel®	hAM	Integra	na	1
AmnioExel®Plus	hACM	Integra	na	1
AmnioFix®	hACM	MiMedx	na	2
AmnioGraft®	hAM	Biotissue	1 to 2	1
AmnioGuard™	hAM	Biotissue	na	1
AmnioWrap™	hACM	Direct Biologics	4	1
Artacent™	hAM	tidesmedical	na	1
BioDGenesis	hP	Derma Sciences	na	1
BioDRestore®	hAM	Integra	na	2
Biovance	hAM	Celularity Inc	4	1
BioXclude®	hACM	Snoasis Medical	2/na	2
Clarix®100	hAM	TissueTech (Amniox Medical)	4	1
Clarix®Cord 1K	hAM; hUC	TissueTech (Amniox Medical)	4/na	4
commercial hAM, not stated which	hACM	not stated	na	1
EpiFix	hACM	MiMedx	na	2
Grafix®Core	hACM	Osiris Therapeutics, Inc	4	1
Grafix®Prime	hAM	Osiris Therapeutics, Inc	4	1
NeoPatch	hACM	CryoLife/Misonix	1	1
NEOX®CORD 1K	hAM; hUC	TissueTech (Amniox Medical)	na	3
Nucel® *	hAM; hAFC	Organogenesis	na	4
NuShield®	hACM	Organogenesis	na	1
Omnigen™	hAM	NuVision Biotherapies Ltd	4	1
REGE pro	hAM	Egyptian Atomic Energy Authority	na	2
ReNu®	hAM; hAFC	Organogenesis	na	3
surgiGRAFTnano	hAM	SynergyBiologics (Integogen LLC)	Early phase 1	1
SURGENEX SurForce® * (placental based tissue matrix) and Predictive Biotech CORECYTE(TM) (hUC-WJ-MSCs)	hAM; hUC-WJ-MSC	SURGENEX & Predictive Biotech, Inc.	na	1
TTAX01	hUC	TissueTech (Amniox Medical)	2 and 3	4
			Total No. trials	48

* Indicates this product can also be found in supplemental table 3.
