# Supplementary material for: Perinatal derivatives application: Identifying possibilities for clinical use
Source: Front Bioeng Biotechnol. 2022 Oct 11;10:977590. doi: 10.3389/fbioe.2022.977590 (PMC9595339; doi:10.3389/fbioe.2022.977590)
Supplement: Supplementary file 1 [file DataSheet1.zip › Supplementary tables and annexes/supplemental table 5.pdf]

Supplemental table 5. Different to-be-commercialised, fluid-based products found in the database.

Fluid	Active substance	Company	Phase	No. of trials
**FlōGraft®**	**hAF**	**Applied Biologics**	**na**	**1**
**Zofin™**	**hAF**	**Organicell**	**1 or 2**	**1**
			**Total No. trials**	**2**
