# Supplementary material for: Perinatal derivatives application: Identifying possibilities for clinical use
Source: Front Bioeng Biotechnol. 2022 Oct 11;10:977590. doi: 10.3389/fbioe.2022.977590 (PMC9595339; doi:10.3389/fbioe.2022.977590)
Supplement: Supplementary file 1 [file DataSheet1.zip › Supplementary tables and annexes/supplemental table 1.pdf]

Supplemental table 1. Distribution of hAM related to ICD-10.

ICD-10	ICD-10 (General name of condition)	No cases	% cases
VII	Eye and adnexa	11	13,9
VIII	Ear and mastoid process	2	2,5
IX	Circulatory system	4	5,1
XI	Digestive system	9	11,4
XII	Skin and subcutaneous	17	21,5
XIII	Musculoskeletal and connective	15	19,0
XIV	Genitourinary	5	6,3
XVII	Congenital malformations	5	6,3
XIX	Injury, poisoning and external causes	11	13,9
	TOTAL CASES	79	
