# Supplementary material for: Perinatal derivatives application: Identifying possibilities for clinical use
Source: Front Bioeng Biotechnol. 2022 Oct 11;10:977590. doi: 10.3389/fbioe.2022.977590 (PMC9595339; doi:10.3389/fbioe.2022.977590)
Supplement: Supplementary file 1 [file DataSheet1.zip › Supplementary tables and annexes/supplemental table 2.pdf]

Supplemental table 2. Use of cell dose in different groups of medical conditions. The number of cells showed refer to the number of cells used in the treatment independently of the bodyweight of the patient.

ICD-10	ICD-10 (General name of condition)	<1M	1M-10M	10M-100M	>100M
I	Infectious and parasitic			1	
II	Neoplasms				
III	Blood and immune				1
IV	Endocrine, nutritional and metabolic		2	3	1
V	Mental and behavioural		1		
VI	Nervous system			4	1
VII	Eye and adnexa		3	2	2
VIII	Ear and mastoid process				
IX	Circulatory system		1	4	2
X	Respiratory system			2	1
XI	Digestive system		1	2	2
XII	Skin and subcutaneous				
XIII	Musculoskeletal and connective		2	15	4
XIV	Genitourinary			4	
XV	Pregnancy, childbirth and puerperium				
XVI	Conditions perinatal period				
XVII	Congenital malformations			1	
XVIII	Abnormal clinical				
XIX	Injury, poisoning and external causes			1	
XX	External causes of morbidity and mortality		1		
XXI	Factores influencing health status				
XXII	Codes special purposes			1	3
	TOTAL CASES	0	11	40	17
