# Supplementary material for: Perinatal derivatives application: Identifying possibilities for clinical use
Source: Front Bioeng Biotechnol. 2022 Oct 11;10:977590. doi: 10.3389/fbioe.2022.977590 (PMC9595339; doi:10.3389/fbioe.2022.977590)
Supplement: Supplementary file 1 [file DataSheet1.zip › Supplementary tables and annexes/supplemental table 3.pdf]

Supplemental table 3. Different to-be-commercialised cell-based products found in the database.

Cells	Active substance	Company	FDA regulated drug	Phase	No. of trials
BX-U001	hUC-MSC	Baylx Inc	yes	1	2
Cellistem ® Lupus	hUC-MSC	Cells for cells	no	2	1
cenplacel-L (PDA-001)	hPC	Celgene Cellular Therapeutics	na	1 and 2	3
Cordstem-ST	hUC-MSC	CHA Bio & Diostech	na	1 to 2	1
CORLICYTE®	hUC-MSC	CellResearch Corporation	yes	1	1
CYNK-001	hPC	Celularity Incorporated	yes	1	1
Cyto-MSC	hUC-MSC	Cytopeutics®	no	1 to 2	1
HB-MSC1	hPMSC	CHU de Reims	no	2	1
MSCTC-0010	hUC-MSC	IMAC Holdings, Inc.	yes	1	1
ORBCEL-C	hUC-MSC	Orbsen Therapeutics,	no	1 to 2	1
PDA-002	hPC	Celgene Corporation has been acquired by Bristol-Myers Squibb (formerly Celularity)	na	1 and 2	2
Pericord (combined product)	hUC-WJ-MSC	Fundació Institut Germans Trias i Pujol	no	1	1
PLX-PAD	hPMSC	Pluristem Therapeutics Inc.	yes	2	1
PLX-R18	hPMSC	Pluristem Therapeutics Inc.	yes	1	1
PrimePro	hUC-MSC	Thomas Advanced Medical LLC	yes	1	1
SCLnow 19#	hUC-MSC	Sclnow Biotechnology	no	1, 1/2 and 2	5
Shanghai Life UC-MSC	hUC-MSC	Shanghai Life Science & Technology	na	1	1
UMC119-01	hUC-MSC	Meridigen Biotech	yes	2 and 3	1
UMC119-06	hUC-MSC	Meridigen Biotech	yes	1	2
UMSC01	hUC-MSC	Ever Supreme Bio Technology Co., Ltd.	yes	1	1
Nucel® *	hAM; hAFC	Organogenesis	no	na	4
SURGENEX SurForce®* (placental based tissue matrix) and Predictive Biotech CORECYTE(TM) (hUC-WJ-MScs)	hAM; hUC-WJ-MSC	SURGENEX & Predictive Biotech, Inc.	https://www.fda.gov/inspections-compliance-enforcement-and-criminal-investigations/warning-letters/predictive-biotech-608322-08172020	na	1
				Total No. trials	34

* Indicates this product can also be found in supplemental table 4.
